# Secretory Vesicles Are Preferentially Targeted to Areas of Low Molecular SNARE Density

**DOI:** 10.1371/journal.pone.0049514

**Published:** 2012-11-15

**Authors:** Lei Yang, Alison R. Dun, Kirsty J. Martin, Zhen Qiu, Andrew Dunn, Gabriel J. Lord, Weiping Lu, Rory R. Duncan, Colin Rickman

**Affiliations:** 1 Institute of Biological Chemistry, Biophysics and Bioengineering, Heriot-Watt University, Edinburgh, United Kingdom; 2 Mathematical and Computer Sciences, Heriot-Watt University, Edinburgh, United Kingdom; Institut Curie, France

## Abstract

Intercellular communication is commonly mediated by the regulated fusion, or exocytosis, of vesicles with the cell surface. SNARE (soluble N-ethymaleimide sensitive factor attachment protein receptor) proteins are the catalytic core of the secretory machinery, driving vesicle and plasma membrane merger. Plasma membrane SNAREs (tSNAREs) are proposed to reside in dense clusters containing many molecules, thus providing a concentrated reservoir to promote membrane fusion. However, biophysical experiments suggest that a small number of SNAREs are sufficient to drive a single fusion event. Here we show, using molecular imaging, that the majority of tSNARE molecules are spatially separated from secretory vesicles. Furthermore, the motilities of the individual tSNAREs are constrained in membrane micro-domains, maintaining a non-random molecular distribution and limiting the maximum number of molecules encountered by secretory vesicles. Together our results provide a new model for the molecular mechanism of regulated exocytosis and demonstrate the exquisite organization of the plasma membrane at the level of individual molecular machines.

## Introduction

Neuronal and endocrine communication is achieved through the orchestrated action of a highly conserved protein machinery [1], [2]. Neurotransmitter, or hormone, containing secretory vesicles, fuse with the plasma membrane, releasing their signal in to the extracellular milieu. Disruption of this process is observed in a growing number of secretion-deficit diseases [3]–[5]. The SNARE protein family are known to actively mediate the fusion of the secretory vesicle and plasma membranes by catalyzing the merger of the two opposing bilayers [1], [2], [6], [7]. The vesicular SNARE (vSNARE), synaptobrevin 2, interacts with the plasma membrane SNARE proteins (target SNAREs or tSNAREs), syntaxin 1 and SNAP-25, forming a highly stable helical complex [6], [8], [9]. The energy liberated through the formation of this complex is thought to provide the driving force for exocytosis. Indeed the SNAREs have been demonstrated to be sufficient to fuse artificial bilayers in vitro, with accessory factors serving to regulate this process [7], [10]–[12].

An emerging theme in membrane biology is the organization of membrane proteins into large scale molecular assemblies [13]. Over the last decade, the spatial organization of the plasma membrane SNAREs has been intensively studied [14]–[18]. Importantly, syntaxin and SNAP-25 differ in their mode of membrane attachment; a single transmembrane helix in the case of syntaxin and post-translational acylation of cysteines for SNAP-25 [19]. Despite this difference, both tSNAREs have been observed to exist in apparent ‘clusters’ using fluorescence microscopy, which are hypothesized to be an important functional entity, providing a localized concentrated pool of tSNAREs to facilitate and enhance bilayer fusion [14]–[17]. However, when interpreting these data it is important to understand the influence of the microscope on the recorded image. Light emitting from a single fluorophore will undergo diffraction through the optics of the microscope, appearing much larger in the recorded image than in the original source. For example the fluorescence signal from a sparse distribution of individual single molecules produces an image that can appear as spots, reminiscent of clusters, with a measured size dependent on the imaging modality used [20]. The size of the recorded signal from a single fluorophore determines the lower limit for the accurate determination of cluster size on a particular microscope. The reported size of the tSNARE clusters has tracked the improvement in resolutions of fluorescence microscopy but has always been observed at the lower limit for accurate size determination. We therefore decided to investigate, with molecular precision, the spatial and dynamic organization of individual SNARE protein molecules, to both increase our understanding of regulated secretion and more generally plasma membrane organization.

## Results

**Figure 1:**
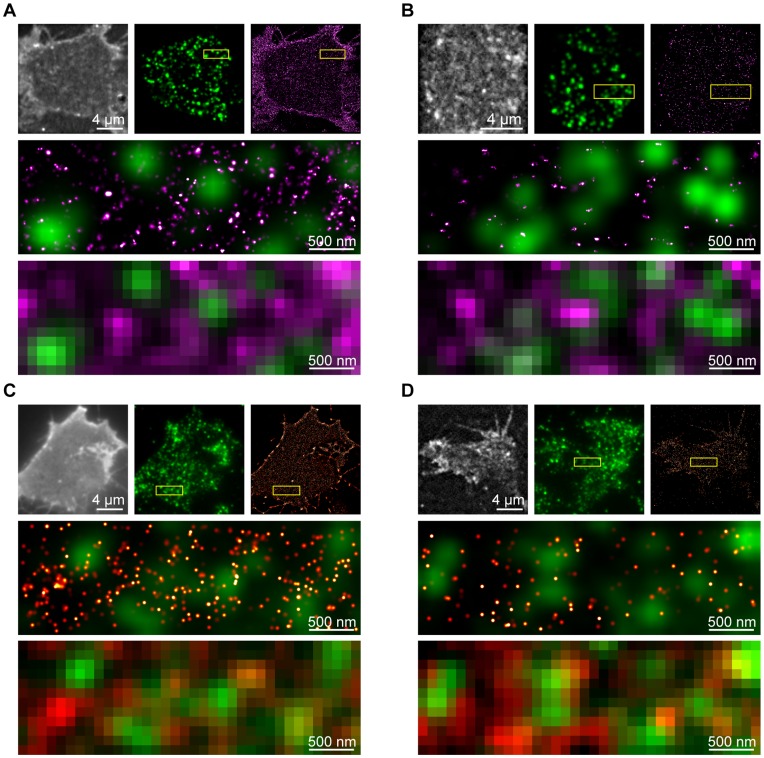
Secretory vesicles preferentially occupy areas of low tSNARE density. (**A**) GSDIM of endogenous SNAP-25. A TIRFM image generated from summation of all detected molecular signals (*upper left*), immunostained vesicles (*upper center*) and rendered GSDIM (*upper right*) are shown for a representative cell. The indicated region (*yellow box*) is shown enlarged (*center*) as an overlay of rendered GSDIM data (*magenta*) and secretory vesicles (*green*). The coordinate data from the GSDIM localization was used to calculate a diffraction-limited resolution TIRFM image of the same field of view (*lower*). (**B**) GSDIM of endogenous syntaxin with panel layout as in (A). (**C**) PALM of SNAP-25. A TIRFM image generated from summation of all detected molecular signals (*upper left*), immunostained vesicles (*upper center*) and rendered PALM (*upper right*) are shown for a representative cell. The indicated region (*yellow box*) is shown enlarged (*center*) as an overlay of rendered PALM data (*red*) and secretory vesicles (*green*). The coordinate data from the PALM localization was used to calculate a diffraction-limited resolution TIRFM image of the same field of view (*lower*). (**D**) PALM of syntaxin with panel layout as in (C).

Recently developed microscopy techniques, including photoactivation localization microscopy (PALM) and ground state depletion microscopy followed by individual molecule return (GSDIM) [21]–[24], can localize individual protein molecules in cells with nanometer certainty. Together, these types of imaging approaches have been termed single molecule localization microscopy (SMLM) [24]. PALM and GSDIM differ in their precise experimental methodology of acquisition, however they both aim to observe sparse sub-sets of a large population of molecules, enabling highly precise localization of each single molecule. SMLM techniques were employed here to probe the spatial organization and dynamics of large cohorts (typically many tens of thousands) of tSNARE molecules on the plasma membrane. Previous studies investigating tSNARE spatial distributions primarily used fluorescent immunostaining, observing clusters at the limit of resolution of their respective approach [14], [25]. Using GSDIM, the nano-scale spatial arrangement of the endogenous tSNARE proteins, syntaxin1 and SNAP-25, was measured on the plasma membrane of neuroendocrine cells (Figure 1A, 1B). These molecules reside in drifts of higher and lower density in the bilayer plane. Surprisingly, in light of current hypotheses regarding tSNARE clusters and their proposed function [14]–[18], vesicles, detected by immunostaining for the calcium sensor synaptotagmin, were located in the areas of low tSNARE density. Analysis of GSDIM is confounded by the repeated localization of the same fluorescent molecule, limiting accurate analysis of molecular density and organization [26]. This makes it impossible to count the number of molecules in a region or comment on any observed clustering. In contrast, PALM has the advantage that each molecule follows a simple linear path of activation, emission and irreversible photobleaching [21], [22]. PALM analysis, using functional fluorescently labeled syntaxin and SNAP-25 [27], [28], reported densities in agreement with previous measures for endogenous tSNAREs [14], [29]. These data recapitulated the findings using GSDIM; the segregation of more dense groups of tSNAREs from sites occupied by secretory vesicles (Figure 1C, 1D). The same organization was observed when cargo was used as the label for secretory vesicles (Figure S1A, S1B). Importantly, the molecular arrangement of the tSNAREs is not representative of a random distribution; instead the molecules conform to a non-random ordered model of organization (Figure S2C, S2D). The molecular map derived by GSDIM and PALM can be used to generate an image, of the same region, as observed using a standard fluorescence microscope or under STED illumination (Figure 1 and Figure S1C–E). At these lower resolutions, partial overlap between tSNARE fluorescence signals and secretory vesicles is apparent, in agreement with previous reports [17], [25].

**Figure 2:**
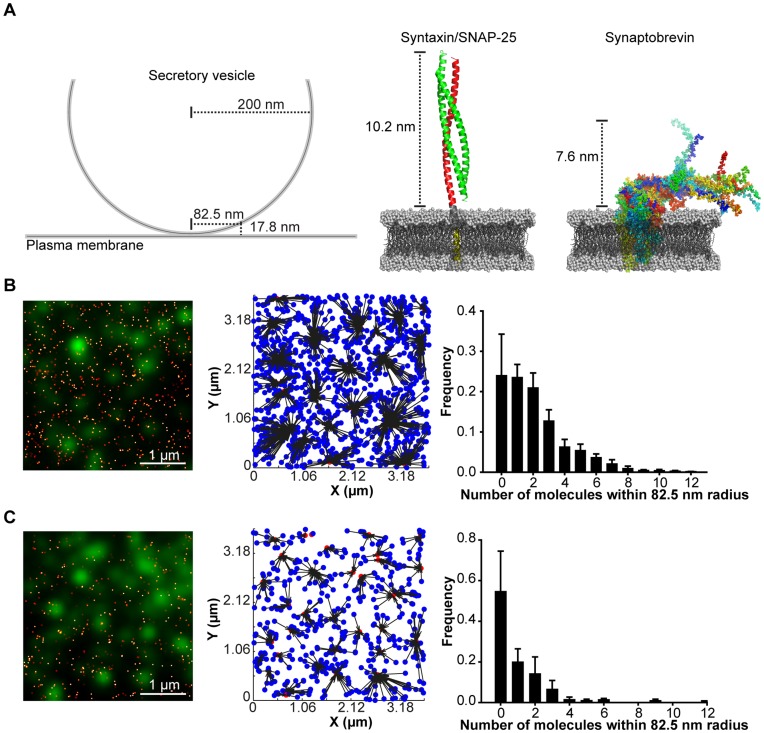
Low numbers of tSNAREs are within a functional distance of secretory vesicles. (**A**) A schematic representation of a secretory vesicle (*left*). A ribbon representation of the complex formed between syntaxin and SNAP-25 (*center*, based on PDB:3IPD [31]) and NMR structures of synaptobrevin (*right*, based on PDB:2KOG [30]) both in synthetic lipid bilayers. The combined maximum reach of these proteins when in opposing bilayers is 17.8 nm. Assuming a distance of 0 nm between opposing bilayers, a chord 17.8 nm from the plasma membrane would have a half-length of 82.5 nm (*left*). This radial distance was used to calculate the number of plasma membrane SNAREs residing under each secretory vesicle. (**B**) A region of plasma membrane showing an overlay of rendered PALM data of SNAP-25 (*red*) and immunostained secretory vesicles (*green*). The center of mass of each secretory vesicle was calculated and used in a nearest neighbor analysis (*center*). The number of SNAP-25 molecules within 82.5 nm of each vesicle was calculated and is shown as a frequency histogram of mean ±SEM (n = 5 cells). (**C**) As in (B) but for PALM localized syntaxin (n = 5 cells).

In addition to the rendered GSDIM and PALM images, the precise X-Y co-ordinates of every molecule are recorded, with a typical localization accuracy of 4–10 nm and 8–21 nm respectively. Lateral drift of the sample can compound single molecule localization approaches. To negate this an adapted sample holder was utilized resulting in a lateral drift of 5 nm in either dimension over the recording period (Figure S2A, S2B). This is of the order of the inaccuracy in the molecular localization in both PALM and GSDIM and hence has no impact on the observed molecular organization. This allows for the quantitative appraisal of tSNARE molecular organization relative to secretory vesicles (also localized with similar nano-scale precision) using nearest neighbor analysis. Available structural information of SNARE proteins in lipid bilayers [30], [31] indicates that the maximum separation over which the plasma membrane and vesicular SNARE proteins could interact is 17.8 nm (Figure 2A). Assuming a zero nanometer distance between the plasma membrane and secretory vesicles (previously used to define ‘docked’ secretory vesicles by electron microscopy [32]) this would provide a maximum radius of 82.5 nm from the center of the secretory vesicle, over which the SNARE proteins would be predicted to be able to interact and drive membrane fusion. This scenario provides the most stringent criteria for measurement. The lateral coordinate data were thus used to assign each molecule to its nearest secretory vesicle and then the number of detected molecules, within 82.5 nm of each vesicle center, was measured (Figure 2B and 2C). This revealed that the average number of each tSNARE, within a functionally relevant distance of each vesicle was of the order of one or two molecules, in good agreement with the estimates of endogenous SNARE molecular density reported by GSDIM.

**Figure 3:**
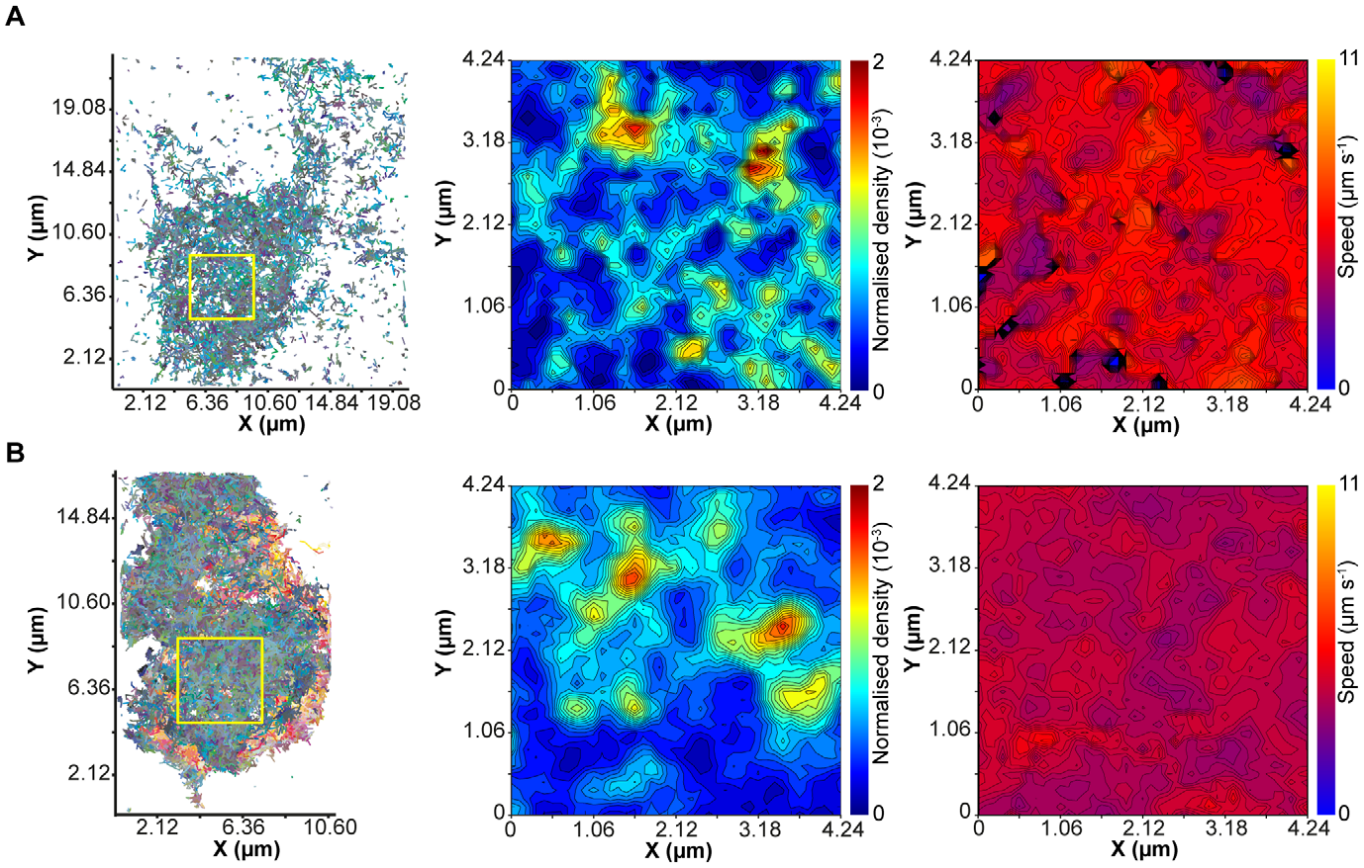
tSNARE molecular dynamics in living membranes. (**A**) Compilation of tracks from individual molecules of SNAP-25 with a temporal resolution of 50 ms(*left*). The indicated region (*yellow box*) was selected and segmented in to an array of 100 nm × 100 nm boxes. The number of tracks passing through each box was measured and is shown as a contour map of normalized density (*center*) such that the sum of all density values is equal to one. The mean speed of all tracks passing through each sampling box was also measured and is displayed as a contour map (*right*). (**B**) As in (A) but for sptPALM of syntaxin.

The previous experiments provide a snapshot of the spatial molecular distribution of the tSNAREs and secretory vesicles with maximum precision, but require immobilization of the protein molecules through fixation [33]. The plasma membrane of a live cell, however, is a highly dynamic environment [34], and so we decided to employ the PALM approach in live cells with single particle tracking (sptPALM) [35]. The sptPALM approach has the advantage of being able to track sparse numbers of protein molecules repeatedly, providing information at the level of individual molecular motion for the whole population of proteins observed (Figure S3A). Approximately 25,000 individual protein molecules were tracked in the basal plasma membrane of each cell, providing nano-scale information on the molecular motion of proteins in live cells with high temporal resolution (Figure 3). The resulting complex network of molecular tracks can be simplified by the generation of ‘contour maps’ reporting the parameters of protein movement in a region of the plasma membrane. sptPALM was performed for both SNAP-25 and syntaxin, both of which exhibit a heterogeneous spatial distribution in their movement with regions of high and low density observed on the plasma membrane (Figure 3A and 3B). This is in agreement with the spatial heterogeneity observed for syntaxin and SNAP-25 using GSDIM and PALM in fixed cells. Speed contour maps of the same region show only small variations in the molecular velocity across the plasma membrane with no apparent correlation with track density.

**Figure 4:**
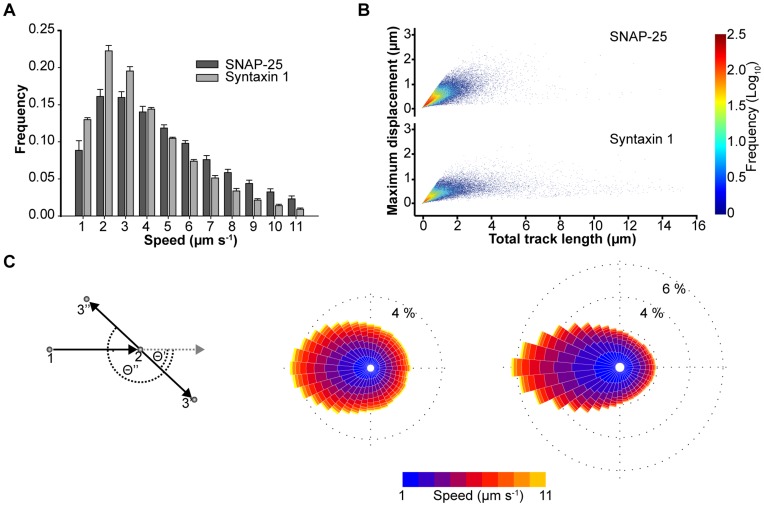
tSNARE molecules exhibit a restricted lateral diffusion in the plasma membrane. (**A**) Combined mean speed for each individual tracks of SNAP-25 and syntaxin shown as a frequency histogram of mean ± SD (n = 3 cells). (**B**) A combined scatter plot of total track length against maximum displacement for SNAP-25 and syntaxin (78641 and 48027 tracks respectively). Maximum displacement has an upper limit of ∼1.6 µm regardless of total track length. (**C**) Analysis of individual track movement demonstrates syntaxin and SNAP-25 tend to reverse direction. A schematic of the analysis applied is shown (*left*). Two points of a track are shown (numbered 1 and 2). Deviation of the third point (3' or 3'') from a forward trajectory (*grey dashed line*) was measured (Θ' or Θ'' respectively) and angle data combined in to a ‘rose diagram’ histogram for SNAP-25 (*center, 197,489 data points*) and syntaxin (*right, 188,916 data points*). For the 36 wedges (each corresponding to 10°), the length indicates the normalize frequency of molecular travel in that direction. Color corresponds to speed.

Both tSNAREs exhibit a single component distribution of mean track speed, with syntaxin having an overall lower mean speed distribution than SNAP-25, as may be expected for an integral membrane protein compared to a peripherally associated molecule (Figure 4A). This speed differential was also confirmed using FRAP (Figure S3B–D). It is important to note that both of these approaches are fit by a single diffusion component and do not discriminate between the molecular motions of different oligomeric tSNARE states. Interestingly, comparing total track length to maximum displacement (the longest distance between any two points in the track) showed that despite the presence of long molecular tracks, both syntaxin and SNAP-25 exhibited maximum displacements of less than 1.6 µm (Figure 4B and Figure S4). This could be indicative of the tSNAREs moving with a caged motion through inclusion in, or exclusion from, domains on the plasma membrane [34], [36]–[38]. To understand this, we examined every individual step in every molecular track (approximately 190,000 events from at least three cells for each tSNARE). Taking pairs of consecutive points, labeled 1 and 2 (Figure 4C) to define the direction of travel, we asked the question: where does a molecule choose to go next? We found that, relative to their current trajectory, the molecules exhibit a distinct bias towards reversing direction (Figure 4C). This is analogous of a ball ricocheting inside a box and indicates that the tSNARE proteins are contained in micro-domains, constraining their molecular motion, and maintaining a non-random spatial distribution.

In addition to the lateral movement of the tSNAREs in the plasma membrane secretory vesicles are also mobile [39], [40]. Electron microscopy (on fixed samples) demonstrated that some secretory vesicles are docked in close apposition to the plasma membrane [32], [40]. Under TIRFM illumination, membrane-proximal vesicles exhibit a highly restricted lateral diffusion often referred to as ‘morphological docking’ [40], [41]. To characterize the molecular motion of secretory vesicles, we tracked vesicles up to the point of fusion (Figure S5A–C), finding that these vesicles exhibited a tethered motion with a relatively slow speed. Immediately prior to exocytosis, a nascent fusing vesicle undergoes a brief rapid acceleration in its movement, in agreement with recent studies in chromaffin cells [39].

**Figure 5:**
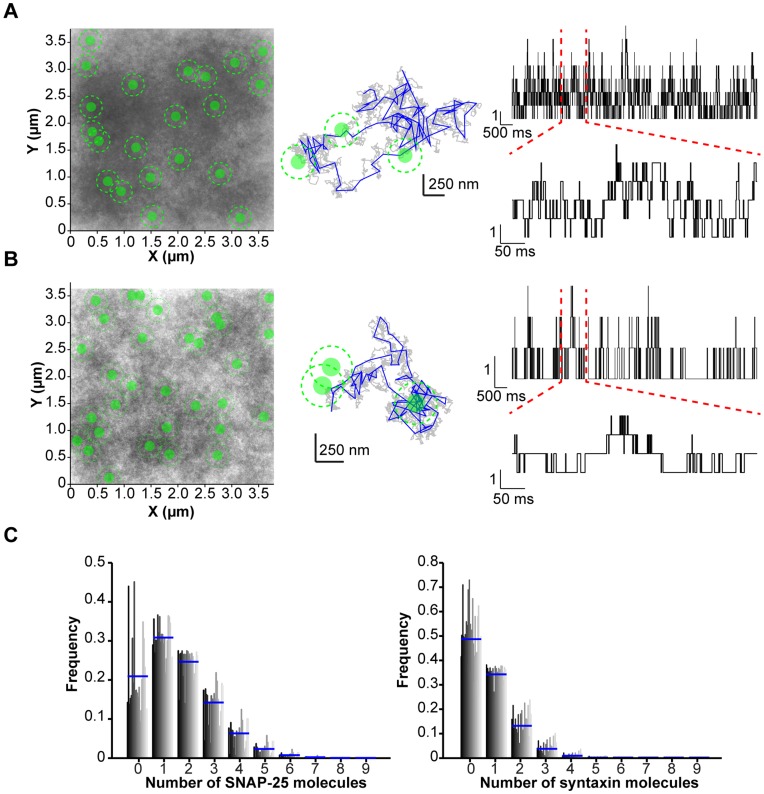
Simulations of the dynamic encounters of secretory vesicles and tSNARE molecules. (**A**) Initial positions of secretory vesicles and SNAP-25 were derived from PALM experiments. Over 5 s molecules and secretory vesicles were allowed to diffuse, bound by the restrictions imposed by the model. A combined image (*left panel*) of all tracks (*grey*) and secretory vesicles (*green, disc signifies sampling window and dashed line circumference of the vesicle*) is shown. A single molecular path is shown (center) with trajectory sampled at 10 µs (*grey*), sampled every 50 ms as in the sptPALM (*blue*) and secretory vesicles (*green*) The number of SNAP-25 molecules within the sampling window is shown over time for a single secretory vesicle over 5 s (*upper right*) and enlarged over 500 ms (*lower right*). (**B**) As in (A) but using initial positions for secretory vesicles and syntaxin derived from PALM experiments (**C**) The probability of each vesicle having a tSNARE within the sampling window at any particular time was calculated using the traces shown in panels a and b. The number of tSNAREs is plotted against frequency for each secretory vesicle in the simulation (*vertical bars, dark grey to light grey*). The mean for all secretory vesicles in each bin is shown (*blue line*). This shows that at any given point in time the majority of vesicles experience three or fewer SNARE molecules.

As it is not possible to record the movement of secretory vesicles and individual tSNARE molecules simultaneously over the same time course, a modeling approach was used to combine the quantified data describing the non-random distribution of the tSNAREs, the molecular dynamics of the individual tSNAREs and the motion of the secretory vesicles into a single unifying simulation. The model was kept as simple as possible, using only parameters derived from sptPALM and vesicle tracking data, along with initial positions for individual tSNAREs and secretory vesicles from fixed cell PALM datasets. A five second simulation was chosen to replicate the maximum period over which a single molecule could be recorded under sptPALM. A single representative simulation for both SNAP-25 and syntaxin is shown (Figure 5A and 5B) along with an enlarged example track. These *in silico* molecules had a resulting speed distribution similar to that measured in sptPALM and produced a comparable distribution for total track length against maximum displacement (Figure S5D, S5E). The simulation allowed the monitoring of the number of mobile tSNAREs within range of interaction with mobile secretory vesicles (Figure 5A, 5B). The numbers of SNARE molecules resident under each secretory vesicle, at any point in time, typically ranged from zero to seven (Figure 5C). This indicates that the tethering and docking of secretory vesicles at the plasma membrane, combined with the restricted freedom of lateral diffusion for the individual SNARE molecules maintains secretory vesicles in a low density SNARE environment.

## Discussion

Using super-resolution microscopy techniques with molecular resolution, we demonstrate that tSNARE molecules exist in a non-random spatial distribution, resulting in areas of low and high molecular density, which give rise to the apparent clusters in diffraction-limited microscope data. Our findings show that, at rest, secretory vesicles do not reside over dense clusters of tSNAREs on the plasma membrane. This is in contrast to current models of SNARE-driven membrane fusion where the secretory vesicle is hypothesised to co-locate with the tSNAREs. However, the majority of studies examining tSNARE clustering have observed, at most, only partial colocalisation with secretory vesicles [17], [25]. Furthermore these studies have been limited to a supra-molecular resolution. Convolution of our SMLM data, using a theoretical point spread function equivalent to a diffraction limited microscope, or STED illumination, recapitulated these observations of partial colocalisation. The areas of low tSNARE molecular density are favourably targeted by secretory vesicles, as sites of docking, by an as yet undefined mechanism.

The spatial organization of the tSNAREs is maintained, in part, by the restricted mobility of tSNAREs, constrained as though in micro-domains. There are a number of proposed mechanisms for protein sequestration in micro-domains through protein-protein or protein-lipid interactions which serve as generalized principles for membrane organization [36]–[38]. Our data support and extend current paradigms of membrane organization by providing quantitative data at the level of very large cohorts of individual molecules and organelles in living cells. Interestingly, the SNARE proteins, studied here, have previously been used extensively as model proteins in such studies [14]–[16]. What is now becoming clear is that the formation and maintenance of the membrane architecture of the tSNAREs is multifactorial, including contributions from both lipidic and protein sources [14]–[16], [25], [42].

What implication does this organization of tSNARE molecules and secretory vesicles have for membrane fusion? It is known that secretory vesicles undergo a rapid movement immediately prior to fusion (Figure S5) [39]. This lateral movement of around 50 nm, however, would be insufficient to move the vesicle on to a denser region of tSNAREs from their tSNARE-sparse starting position. Instead this movement may be simply a result of the zippering of SNARE complexes off-center from the axis of the secretory vesicle, resulting in the small rapid translation observed. The low number of tSNAREs close to the secretory vesicle should therefore be sufficient to drive membrane fusion. Indeed the number of tSNAREs observed immediately adjacent to the secretory vesicles falls within the range reported to be sufficient to drive membrane fusion in a variety of biophysical experiments [43]–[47]. Clearly secretory vesicles residing in a region of the plasma membrane with insufficient numbers of tSNAREs would be unable to fuse. Conversely, above this lower threshold, the probability of vesicle fusion would, in part, be determined by the number of tSNAREs in close proximity to the secretory vesicles. By regulating the tSNARE molecular landscape, through one or more candidate mechanisms [15], [16], [18], [25], the cell could dynamically modulate individual release probabilities and thereby the kinetics of the cellular response.

## Materials and Methods

### Cell Culture and Immunofluorescence

Plasmids encoding PACherry-SNAP25_1–206_, EGFP-SNAP25_1–206_, PACherry-Syntaxin1a_1–288_, EGFP-Syntaxin1a_1–288_ and NPY-EGFP were described previously [18], [48]. PC-12 cells were maintained and propagated as described [18]. For microscopy, coverslips were extensively cleaned in a sonicating waterbath containing 0.1 M sodium hydroxide and 0.1% Decon-90 for 30 seconds followed by washing in deionized water, ethanol and acetone. Coverslips were then coated in 100 µg/ml poly-D-lysine (Sigma) prior to seeding with PC-12 cells (ATCC). Cells were transfected 24 hours after plating using Lipofectamine (Invitrogen) and left for a further 48 hours prior to use in experiments. Immunostaining was performed as described previously with extensive fixation in 4% paraformaldehyde solution for 1 hour at room temperature to ensure maximal immobilization of cellular proteins [33]. For immunostaining, syntaxin-1A was detected using the monoclonal antibody HPC-1 (Sigma), SNAP-25 using the monoclonal antibody SMI81 (Sternberger monoclonals) and secretory vesicles using a polyclonal anti-syntaptotagmin antibody (SySy). Antibodies were detected by immunofluorescence using immunoglobulin Fab’ fragments labeled with Alexa 488 or Alexa 647 (Invitrogen).

### Microscope Setup

All experiments were performed on an inverted IX81 microscope (Olympus) using a 150 × 1.45 NA objective. Illumination was provided by a xenon-mercury lamp or a fully motorized four laser TIRF combiner coupled to 405 nm, 491 nm, 561 nm and 540 nm 100 mW lasers. This allowed for rapid switching of penetration depth from widefied to TIRF illumination during experiments. The sample was maintained in an environmental chamber (Okolabs) at 21°C for fixed samples or at 37°C in 5% CO2, 95% Air for live cells. To minimize lateral drift during acquisition a nosepiece stage (Olympus) was employed. Lateral drift using this stage was ∼±6 nm over a typical 30 minute acquisition. This meant no correction for drift (e.g. using fiducidal markers) was required post acquisition. This is comparable to the localization accuracy, due to the signal to noise ratio of detected single molecules, of 4–21 nm for PALM or GSDIM datasets. Fluorescence emission was detected using a 512×512 pixels, water-cooled EMCCD camera (Hamamatsu).

### Single Molecule Localization Microscopy

GSDIM microscopy was performed based on previously described methods [26]. Cells were fixed and immunostained as above. To ensure efficient switching of Alexa 647, cells were imaged in 0.5 mg/ml glucose oxidase, 40 µg/ml catalase, 10% wt/vol glucose and 50 mM β-mercaptoethylamine. In a typical experiment, cells were initially excited by 491 nm laser light under TIRF illumination to acquire Alexa 488 labeled vesicle fluorescence. The cell was then continuously illuminated with 640 nm laser light under TIRF illumination for 15 to 30 minutes. Emitted fluorescence was detected using an EMCCD camera with an EM Gain of 100–400 and a frame rate of 20 Hz. The resulting image sequences were subsequently analyzed using single molecule identification and localization algorithms described below. The repeated cycling of fluorophores between the excited and dark states results in repetitive localization of the same fluorophore multiple times.

PALM microscopy was performed based on previously described methods [21], [22]. Cells, expressing photoactivatable mCherry labeled SNAREs were fixed, and immunostained as required, as detailed above. Cells were imaged in PBS at 21°C. In a typical experiment, cells were initially excited by 491 nm laser light under TIRF illumination to acquire Alexa 488 or GFP labeled vesicle fluorescence. Photoactivatable mCherry was then activated with a brief pulse (1 to 250 ms) of 405 nm laser light under TIRF illumination followed by acquisition of 20 to 40 frames using a 561 nm laser under TIRF illumination and an EMCCD camera with an EM Gain of 400–600 at 5 Hz. This cycle of activation and acquisition was repeated between 150 and 300 times with the activation pulse duration increasing gradually during the experiment.

For static PALM and GSDIM datasets single molecules were detected using a Matlab routine kindly provided by Samuel Hess (Maine) [22]. Long-lived dark states can result in the repeated localization of the same fluorophore in PALM experiments (particularly with mEos2 and Dronpa) [49]. To minimize any influence of dark states in our data, individual frames between activation pulses were summed together using ImageJ before localization. Localized datasets were then used for further analysis in Matlab, or rendered at high resolution. Rendering of localized molecules was performed using the same Matlab algorithms and false colored in ImageJ.

### sptPALM

Cells, expressing photoactivatable mCherry labeled SNAREs were imaged in phenol red free culture medium at 37°C and 5% CO_2_, 95% air. Photoactivatable mCherry was activated with a brief pulse (1 to 40 ms) of 405 nm laser light under TIRF illumination followed by acquisition of 100 frames using a 561 nm laser under TIRF illumination and an EMCCD camera with an EM Gain of 600–800 at 20 Hz. This cycle of activation and acquisition was repeated between 150 and 300 times with the activation pulse duration increasing gradually during the experiment.

An automated particle detection and tracking system has been developed and applied [50]. The system combines particle detection in each single image frame and frame-to-frame particle correspondence implemented in Matlab. Particle detection in each single frame comprises three components: (1) particle probability image mapping [51], (2) refinement of particle probability image, and (3) particle segmentation. The first component is implemented by three steps: (a) The Haar-like feature for each pixel is measured in the original grayscale image; (b) A weak threshold is applied to the Haar-like feature to coarsely classify each pixel into one of two classes: particle or background; (c) A particle probability concept is defined as the ratio of the number of spatially connected particle pixels to the total number of pixels in a small region of a particle size. Particle features are significantly enhanced in the particle probability image. The second component is implemented by applying a rotationally symmetric Gaussian low pass filter to the newly obtained particle probability image to get more accurate particle probability at each pixel. The third component is implemented by firstly estimating particles existing regions and their corresponding markers of particles from the refined particle probability image, and then using the marker-controlled watershed transform to accurately segment the particle regions from the original grayscale image. Our particle detection algorithm allows for the detection of particle positions at sub-pixel level and accurate estimation of particle topologies such as size and intensity. The robust frame-to-frame particle correspondence is finally implemented by incorporating these particle topologies into the system state vector of an Interacting Multiple Model (IMM) filter to better deal with particle motion modeling and robust data association. Here three motion models, random walk, first order and second order linear extrapolations are used for motion modeling, and a dynamic programming algorithm is used to optimize the particle correspondence by minimizing the association cost function.

### Vesicle Tracking and Fusion

For vesicle tracking and stimulation experiments, PC12 cells, expressing NPY-EGFP, were maintained on the microscope at 37°C and 5% CO_2_, 95% air. Cells were imaged in KREBs Buffer (115 mM Sodium Chloride, 5 mM Potassium Chloride, 24 mM Sodium Bicarbonate, 2.5 mM Calcium Chloride, 1 mM Magnesium Chloride, 10 mM HEPEs (pH 7.4), 0.1% (w/v) BSA) adjusted to 290 mOsM. For stimulation, ATP was added during the recording to a final concentration of 300 mM. Secretory vesicle movement and fusion were acquired using a 491 nm laser under TIRF illumination and an EMCCD camera with an EM Gain of 200–400 at 20 Hz.

To determine the mobility of secretion competent vesicles, high-speed image sequences were acquired as detailed above. Vesicles undergoing fusion were identified by the characteristic rapid increase in fluorescence upon EGFP un-quenching and then the exponential decay resulting from diffusion of cargo molecules from the site of fusion. Small regions of interest were excised surrounding these fusion events containing the preceding frames. These single vesicle movies were then subjected to particle tracking using Imaris (Bitplane) aligning the maximum intensity frame, equating to fusion, as the final frame to allow the averaging of multiple events.

### FRAP

Cells expressing EGFP labeled SNAREs were imaged in phenol red free culture medium at 37°C and 5% CO_2_, 95% air. Fluorescence recovery after photobleaching was carried out using the Olympus Cel?FRAP hardware attachment in conjunction with TIRF illumination. A circular bleach area of radius 0.742 µm was selected and bleached in the camera dead time between frames 5 and 6 of a total image train of 30 acquired at 32 Hz. Membrane sheets were prepared by sonication as described previously [25]. In brief, cells were grown on coverslips as standard. The coverslip was immersed in 100 mL of sheet sonication buffer (120 mM potassium glutamate, 20 mM potassium acetate, 10 mM EGTA, 4 mM MgCl_2_, 2 mM ATP, 0.5 mM dithiothreitol, and 20 mM HEPES-KOH, pH 7.2) in a 9 cm diameter beaker. A 2 mm sonication probe placed at a height of 1 cm above the coverslip and operated at 40% for 10 s.

Image J was used to extract intensity data from the resulting image files and the software program FRAP_Analyser was used to extract a diffusion coefficient, *D*, from the data gathered from each individual FRAP experiment.

### SMLM Spatial Analysis

Following single molecule localization the spatial distribution of individual molecules was analyzed from the coordinate information. Ripley’s analyses were performed using custom written Matlab algorithms. To compare the observed spatial distribution to the random state, the same numbers of molecules, in the same spatial area, were redistributed randomly 1000 times. For each simulation the Ripley’s K function and L transformation were derived. This is presented as light grey envelopes for the randomized simulations with the test case in black. Deviation of the test case above the envelopes at short radii indicates a non-random morphology with areas of high and low density. Deviation of the test case below the envelopes would indicate some form of minimum distance between adjacent molecules.

To analyze the spatial distribution of secretory vesicles relative to the SNARE molecules, nearest neighbor analysis was performed. Using the PALM coordinates of SNARE proteins and the centroid coordinates of secretory vesicles, SNARE molecules were assigned to their nearest vesicle using a nearest neighbor routine in Matlab. A sampling radii was determined based on the range over which the tSNAREs and vSNARE would be able to interact using available structural information. Following allocation of molecules to their nearest secretory vesicle the number of molecules within 82.5 nm of the centroid of each vesicle was determined.

### Molecular Modeling

The motion of the syntaxin and SNAP-25 molecules were both modeled by Brownian motion with the only free parameter being the noise intensity. This parameter was fixed for each molecule by comparison to the experimental data of speeds and track lengths. Brownian motion is consistent with a small molecule moving under random external forcing. For the vesicles the noise intensity for the Ornstein-Uhlenbeck (OU) process was fixed by comparison to experimental data for the speeds and the mean position was fixed from the PALM datasets. The OU process is consistent with a large molecule undergoing random fluctuations with friction. It describes the caged motion observed experimentally and maintains the non-random spatial distribution. These stochastic equations were solved numerically using the standard Euler–Maruyama method with a time step much smaller than the experimental sample rate of 50 ms. For this simple model no interaction was included between any of the molecules. Initial positions of the molecules were taken from experimental PALM datasets. To investigate the number of tSNARE molecules in range of a vesicle we took a computational domain with periodic boundaries.

## Supporting Information

Figure S1 **Molecular organization of the plasma membrane SNARE machinery.** (**A**) PALM of photoactivatable mCherry labeled SNAP-25. A TIRFM image generated from summed individual molecules (*upper left*), NPY-EGFP labeled vesicles (*upper center*) and rendered PALM (*upper right*) are shown for a representative cell. The indicated region (*yellow box*) is shown enlarged (*center*) as an overlay of rendered PALM data (*red*) and secretory vesicles (*green*). (**B**) PALM of photoactivatable mCherry labeled syntaxin with panel layout as in (A). SMLM datasets can reproduce SNARE clusters observed by diffraction limited optical microscopy and STED. (**C**) GSDIM of endogenous SNAP-25. A TIRFM image of immunostained vesicles (*upper left*) and rendered GSDIM (*lower left*) are shown for a representative cell. The indicated region (*yellow box*) is shown enlarged (*right*) as an overlay of rendered GSDIM data (*magenta*) and secretory vesicles (*green*). This region was convolved to show this region under standard and STED resolutions. (**D**) GSDIM convolved with a standard PSF (*upper left*) and immunostained vesicles (*lower left*). The same region is shown overlaid and enlarged (*right*). The pixel size equates to 106 nm (a 150×1.45 NA objective coupled with a 16 µm pixel detector). (**E**) As in (D) but using a calculated PSF under STED illumination. The pixel size is 30 nm as used in previous publications.
(TIF)

Figure S2 **Spatial analysis of plasma membrane SNARE distributions observed by PALM.** (**A**) To minimize lateral drift a nose-piece stage (Olympus) was employed. The sample chamber was placed on the top plate. The whole microscope was contained within an incubation chamber to minimize air currents and temperature fluctuations. (**B**) 100 nm beads were imaged for 30 minutes at 1 Hz (*upper panel*) and localized by fitting of a 2-dimensional Gaussian distribution to calculate the centroid. The calculated centroid of two beads for each frame in the image train is shown as a scatter plot (*red and green spots*). 99.9% of the points fall within a circle of 6 nm radius. This movement is comparable to the level of accuracy of localization in PALM and GSDIM datasets. (**C**) The coordinates of individual SNAP-25 molecules are plotted (*left panel, blue circles*). The region indicated (*red box*) is shown expanded (*center panel*). Ripley’s K function followed by transformation to derive the L function is shown (*right panel, black line*). The data was randomized 1000 times, maintaining the same area and number of molecules and the L function calculated (*grey lines*). (**D**) As in (C), but using syntaxin PALM coordinate data. Deviation above the random simulations at short sampling distances, as observed in both cases here, indicates a non-random, heterogeneous distribution of areas of higher density reminiscent of clustering.
(TIF)

Figure S3 **Measurement of tSNARE mobility on the plasma membrane.** (**A**) An automated particle detection and tracking system for sptPALM. A flow diagram representing the individual steps is shown. A raw image (part of a large image series) is subjected to automated particle detection. Individual particles are tracked over 100 individual frames and accumulated. This cycle is then repeated for between 160 and 240 individual activation cycles. FRAP measurement of t-SNARE motion in intact cells and membrane sheets. (**B**) Representative frames from a single FRAP experiment on a PC12 cell expressing GFP-SNAP25 (*green*). Photobleaching of a circle of radius 0.742 nm was carried out between frames 5 and 6, and frame 6 - the ‘bleach moment’ - is considered as t = 0. (**C**) Average normalized fluorescence recovery curves from intact cells (*black circles*) and membrane sheets (*grey circles*) for SNAP25 (*left panel*) and syntaxin-1A (*right panel*). Error bars represent standard errors in the mean, n = 3. (**D**) Mean velocities for SNAP25 and syntaxin 1A in intact PC12 cell membranes (*black bars*) and membrane sheets (*grey bars*) extracted from curves fit to normalized FRAP data.
(TIF)

Figure S4 **Analysis of individual movement steps demonstrates syntaxin and SNAP-25 move randomly.** (**A**) A schematic of the analysis applied is shown (*left*). Two points of a track are shown (numbered 1 and 2). The angle of movement was measured (Θ) as shown, and combined in to a rose diagram histogram for SNAP-25 (*center, 287,352 events*) and syntaxin (*right, 257,084 events*). The size of each wedge (corresponding to 10°) indicates the propensity of direction with color corresponding to the speed of the molecule. (**B**) The cumulative number of tracked tSNARE particles against maximum displacement for different total track lengths are shown for SNAP-25 (*left*) and syntaxin (*right*).
(TIF)

Figure S5 **Modeling of the secretory machinery.** Motion of secretory vesicles prior to exocytosis. (**A**) Single frame from an image sequence of a PC-12 cell expressing NPY-EGFP (*left panel*). Individual vesicles were tracked over time and the path color-coded according to their position during the image sequence (*right panel, color scale blue-red-yellow*). (**B**) Secretory vesicles undergoing fusion were detected and the tracked speed plotted over time with 0 sec corresponding to the fusion event. Mean and error bars representing the SEM are plotted (n = 9 vesicles). (**C**) A representative vesicle showing the track up to the point of fusion (*left, orange*). The dashed line indicates the circumference of the secretory vesicle. The speed and intensity of this vesicle is shown over time (*right panel*). (**D**) A speed histogram of SNAP-25 and syntaxin from ten realizations of the simulation is in good agreement with experimentally measured speeds. (**E**) A combined scatter plot of total track length against maximum displacement for SNAP-25 and syntaxin from ten realizations of the simulation. Maximum displacement was defined as the maximum distance between any two points in a track. The limit of maximum displacement is comparable to that observed for sptPALM.
(TIF)
