# Supplementary figures and images for: Secretory Vesicles Are Preferentially Targeted to Areas of Low Molecular SNARE Density
Source: PLoS One. 2012 Nov 15;7(11):e49514. doi: 10.1371/journal.pone.0049514 (PMC3499460; doi:10.1371/journal.pone.0049514)

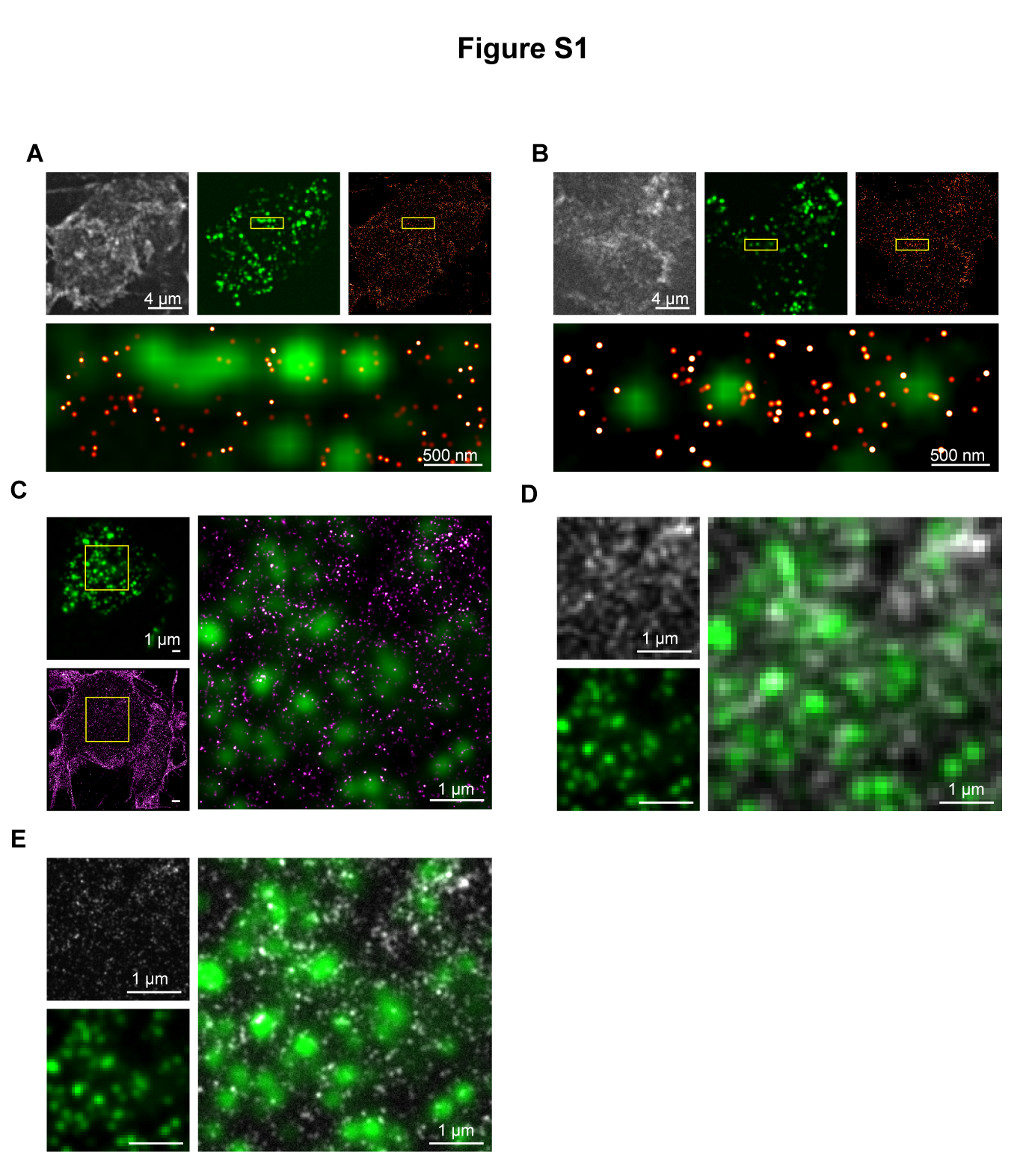

Supplement: Figure S1 — Molecular organization of the plasma membrane SNARE machinery. (A) PALM of photoactivatable mCherry labeled SNAP-25. A TIRFM image generated from summed individual molecules (upper left), NPY-EGFP labeled vesicles (upper center) and rendered PALM (upper right) are shown for a representative cell. The indicated region (yellow box) is shown enlarged (center) as an overlay of rendered PALM data (red) and secretory vesicles (green). (B) PALM of photoactivatable mCherry labeled syntaxin with panel layout as in (A). SMLM datasets can reproduce SNARE clusters observed by diffraction limited optical microscopy and STED. (C) GSDIM of endogenous SNAP-25. A TIRFM image of immunostained vesicles (upper left) and rendered GSDIM (lower left) are shown for a representative cell. The indicated region (yellow box) is shown enlarged (right) as an overlay of rendered GSDIM data (magenta) and secretory vesicles (green). This region was convolved to show this region under standard and STED resolutions. (D) GSDIM convolved with a standard PSF (upper left) and immunostained vesicles (lower left). The same region is shown overlaid and enlarged (right). The pixel size equates to 106 nm (a 150×1.45 NA objective coupled with a 16 µm pixel detector). (E) As in (D) but using a calculated PSF under STED illumination. The pixel size is 30 nm as used in previous publications. (TIF) [file pone.0049514.s001.tif]

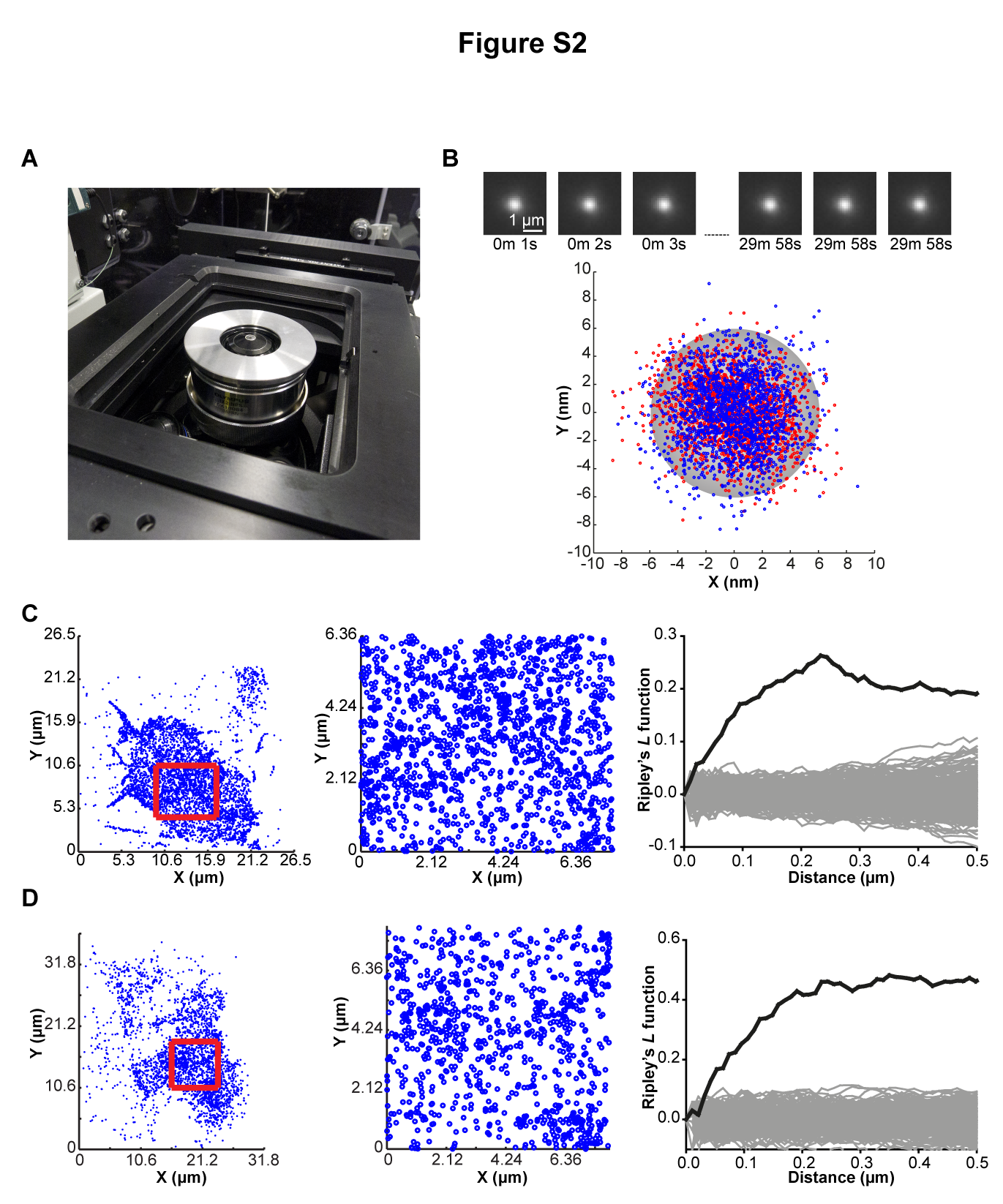

Supplement: Figure S2 — Spatial analysis of plasma membrane SNARE distributions observed by PALM. (A) To minimize lateral drift a nose-piece stage (Olympus) was employed. The sample chamber was placed on the top plate. The whole microscope was contained within an incubation chamber to minimize air currents and temperature fluctuations. (B) 100 nm beads were imaged for 30 minutes at 1 Hz (upper panel) and localized by fitting of a 2-dimensional Gaussian distribution to calculate the centroid. The calculated centroid of two beads for each frame in the image train is shown as a scatter plot (red and green spots). 99.9% of the points fall within a circle of 6 nm radius. This movement is comparable to the level of accuracy of localization in PALM and GSDIM datasets. (C) The coordinates of individual SNAP-25 molecules are plotted (left panel, blue circles). The region indicated (red box) is shown expanded (center panel). Ripley’s K function followed by transformation to derive the L function is shown (right panel, black line). The data was randomized 1000 times, maintaining the same area and number of molecules and the L function calculated (grey lines). (D) As in (C), but using syntaxin PALM coordinate data. Deviation above the random simulations at short sampling distances, as observed in both cases here, indicates a non-random, heterogeneous distribution of areas of higher density reminiscent of clustering. (TIF) [file pone.0049514.s002.tif]

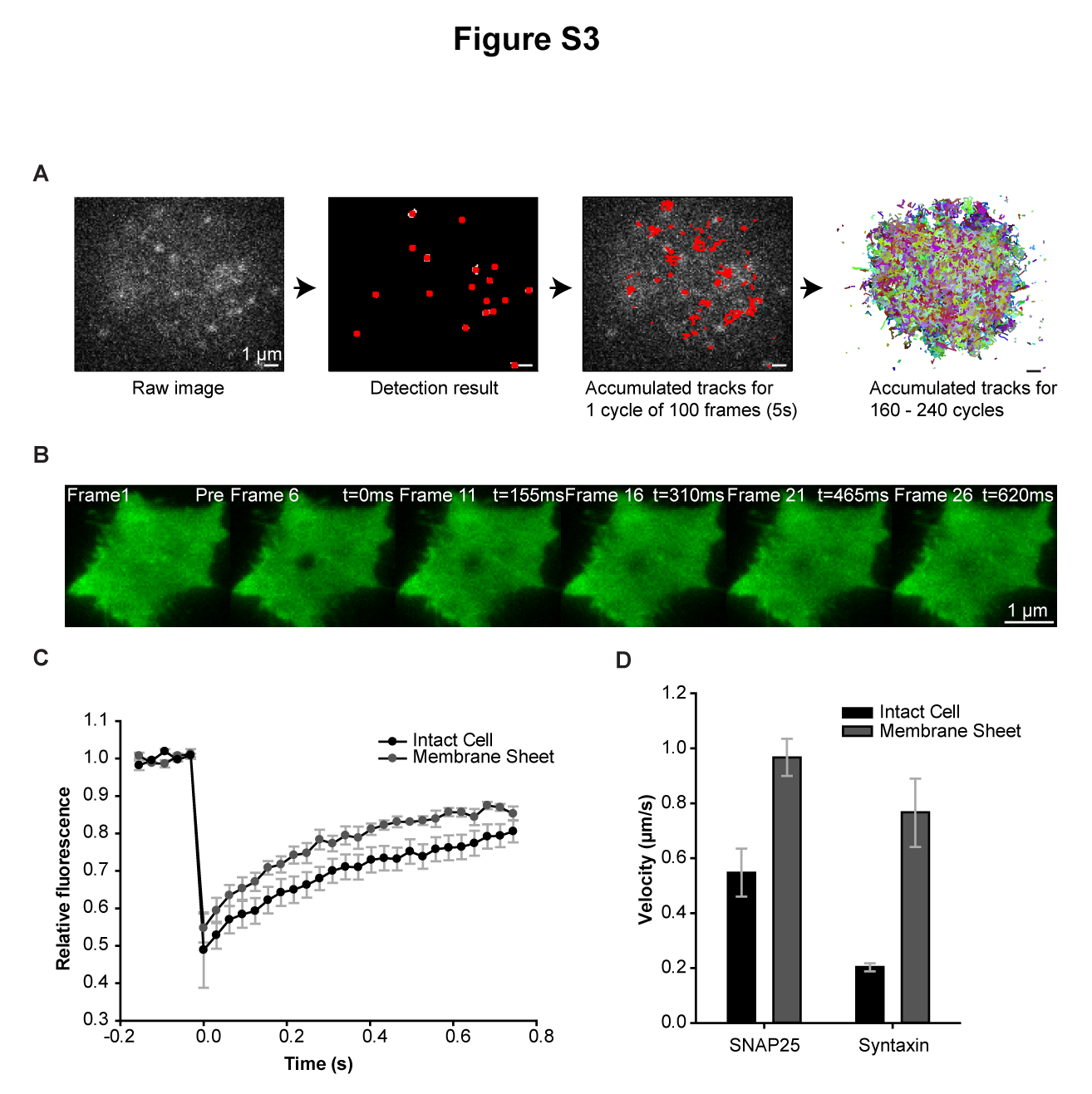

Supplement: Figure S3 — Measurement of tSNARE mobility on the plasma membrane. (A) An automated particle detection and tracking system for sptPALM. A flow diagram representing the individual steps is shown. A raw image (part of a large image series) is subjected to automated particle detection. Individual particles are tracked over 100 individual frames and accumulated. This cycle is then repeated for between 160 and 240 individual activation cycles. FRAP measurement of t-SNARE motion in intact cells and membrane sheets. (B) Representative frames from a single FRAP experiment on a PC12 cell expressing GFP-SNAP25 (green). Photobleaching of a circle of radius 0.742 nm was carried out between frames 5 and 6, and frame 6 - the ‘bleach moment’ - is considered as t = 0. (C) Average normalized fluorescence recovery curves from intact cells (black circles) and membrane sheets (grey circles) for SNAP25 (left panel) and syntaxin-1A (right panel). Error bars represent standard errors in the mean, n = 3. (D) Mean velocities for SNAP25 and syntaxin 1A in intact PC12 cell membranes (black bars) and membrane sheets (grey bars) extracted from curves fit to normalized FRAP data. (TIF) [file pone.0049514.s003.tif]

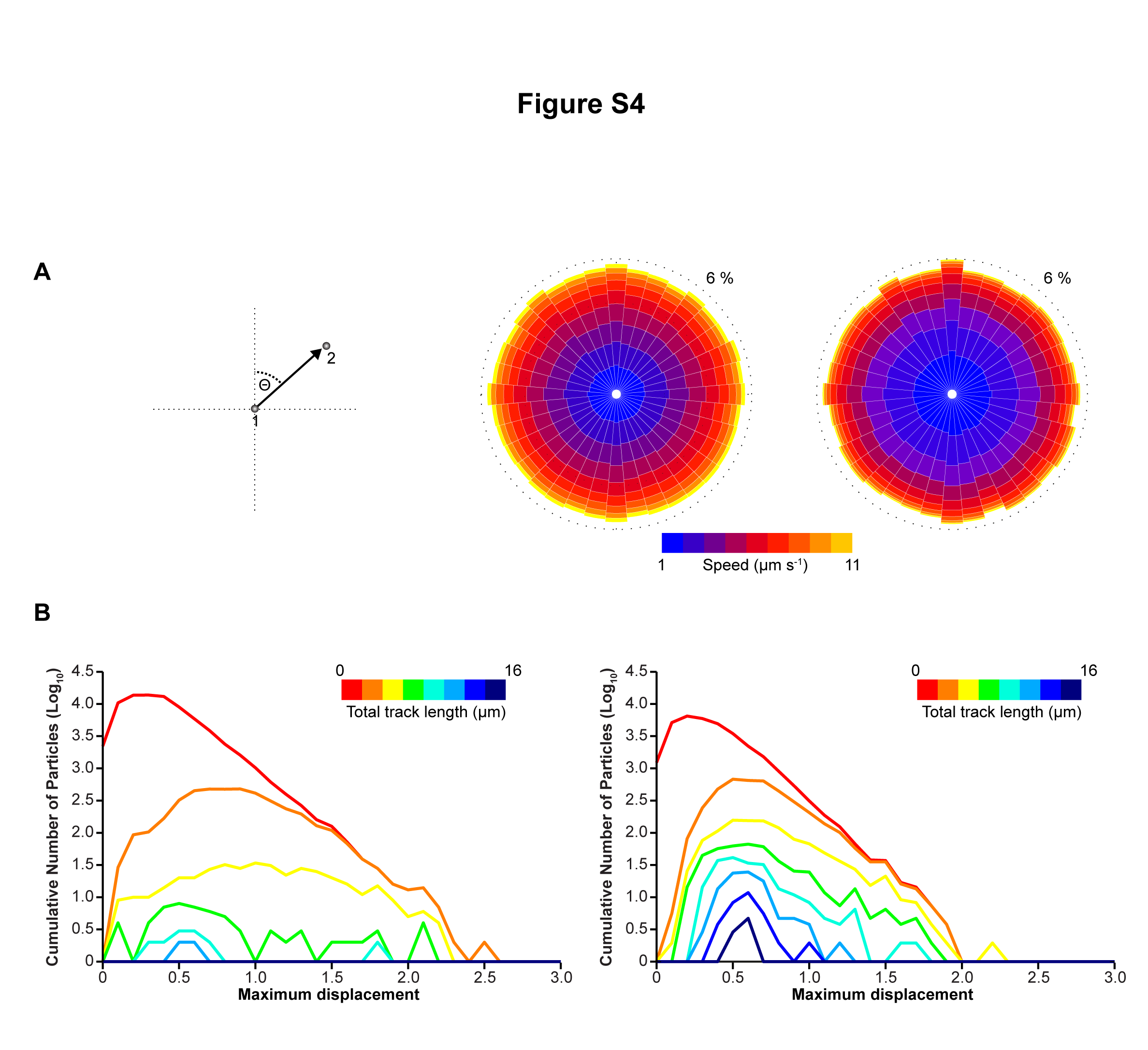

Supplement: Figure S4 — Analysis of individual movement steps demonstrates syntaxin and SNAP-25 move randomly. (A) A schematic of the analysis applied is shown (left). Two points of a track are shown (numbered 1 and 2). The angle of movement was measured (Θ) as shown, and combined in to a rose diagram histogram for SNAP-25 (center, 287,352 events) and syntaxin (right, 257,084 events). The size of each wedge (corresponding to 10°) indicates the propensity of direction with color corresponding to the speed of the molecule. (B) The cumulative number of tracked tSNARE particles against maximum displacement for different total track lengths are shown for SNAP-25 (left) and syntaxin (right). (TIF) [file pone.0049514.s004.tif]

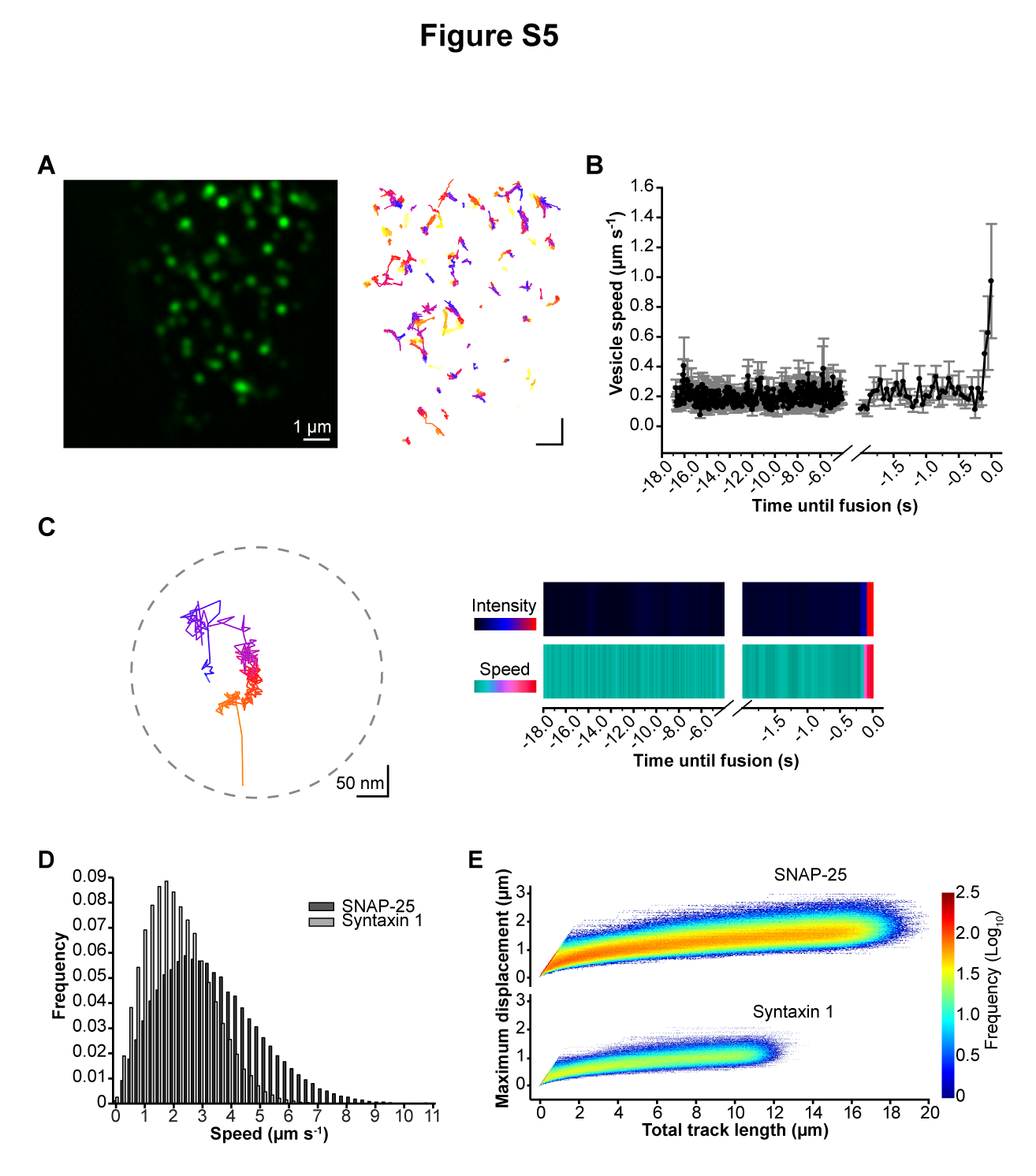

Supplement: Figure S5 — Modeling of the secretory machinery. Motion of secretory vesicles prior to exocytosis. (A) Single frame from an image sequence of a PC-12 cell expressing NPY-EGFP (left panel). Individual vesicles were tracked over time and the path color-coded according to their position during the image sequence (right panel, color scale blue-red-yellow). (B) Secretory vesicles undergoing fusion were detected and the tracked speed plotted over time with 0 sec corresponding to the fusion event. Mean and error bars representing the SEM are plotted (n = 9 vesicles). (C) A representative vesicle showing the track up to the point of fusion (left, orange). The dashed line indicates the circumference of the secretory vesicle. The speed and intensity of this vesicle is shown over time (right panel). (D) A speed histogram of SNAP-25 and syntaxin from ten realizations of the simulation is in good agreement with experimentally measured speeds. (E) A combined scatter plot of total track length against maximum displacement for SNAP-25 and syntaxin from ten realizations of the simulation. Maximum displacement was defined as the maximum distance between any two points in a track. The limit of maximum displacement is comparable to that observed for sptPALM. (TIF) [file pone.0049514.s005.tif]
